# Crimean-Congo hemorrhagic fever: Strategies for diagnosis at initial admission

**DOI:** 10.1016/j.bjid.2025.104516

**Published:** 2025-02-22

**Authors:** Ahmet Melih Şahin, Emrullah Ataş, Sinan Çetin

**Affiliations:** Giresun University, Medical Faculty, Department of İnfectious Diseases and Clinical Microbiology, Giresun, Turkey

**Keywords:** CCHF, Tick bite, Viral hemorrhagic fever, Zoonotic disease

## Abstract

Crimean-Congo Hemorrhagic Fever (CCHF) is a viral hemorrhagic fever common in many regions of the world. There are many diseases in the differential diagnosis of CCHF. In our study, we aimed to predict the diagnosis of CCHF at the time of initial presentation by using clinical and laboratory findings in patients with a preliminary diagnosis of CCHF. In our study, 74 patients with a definitive diagnosis of CCHF and 43 patients with a preliminary diagnosis of CCHF but not diagnosed with CCHF were compared in terms of demographic, clinical and laboratory findings. Multivariate logistic regression analysis and Receiver Operating Characteristics (ROC) curve were used to determine variables to predict the diagnosis of CCHF. Living in an endemic area, tick bite, fever, CRP below 48 mg/L and PCT below 0.52 ng/mL were determined as independent risk factors for CCHF diagnosis. The specificity for cut off values of 2485 mm^3^ for WBC and 970 mm^3^ for neutrophil count were 86 % and 93 %, respectively. The sensitivity for cut off values of 48 mg/L for CRP and 0.52 ng/mL for PCT were 90.5 % and 82.4 %, respectively. In-hospital and 28-day mortality were higher in the non-CCHF group. The differential diagnosis of CCHF is important for planning appropriate isolation procedures and treatments for patients. Additionally, by excluding CCHF, it allows for the early consideration of other diseases in the non-CCHF group that show high mortality. In patients living in endemic areas with tick bites and clinical findings compatible with CCHF, easily accessible tests such as WBC, neutrophil count, CRP and PCT, within the cut-off values identified in our study, will assist in diagnosing CCHF at the initial presentation.

## Introduction

Crimean-Congo Hemorrhagic Fever (CCHF) is the most common viral hemorrhagic fever seen in Eastern and Southern Europe, the Mediterranean, northwestern China, Central Asia, Africa, the Middle East and the Indian subcontinent.^1^ The disease, which spreads to humans through infected tick bites or infected animal blood, is considered endemic in Turkey.^1^ It should be kept in mind that this disease, which often has a subclinical course (88 %), is potentially mortal.^2^ While the case-fatality rate has been reported to be approximately 5 % in our country, this rate has been reported to be 1040 % in different countries according to World Health Organization (WHO) data.^2^, ^3^, ^4^ Considering that a significant proportion of patients do not mention tick bite or tick contact (31.1 %) and the initial symptoms are similar to some febrile diseases, many diseases should be considered in the differential diagnosis of CCHF.^5^, ^6^, ^7^ For the definitive diagnosis of the disease, IgM tests against Crimean-Congo Hemorrhagic Fever Virus (CCHFV) by Enzyme-Linked Immunosorbent Assay (ELISA) or CCHFV RNA tests by Polymerase Chain Reaction (PCR) are required. The fact that these tests are performed in the national central reference laboratory and that obtaining results takes days delays the diagnosis. This may lead to problems in the evaluation of all diseases in the differential diagnosis, patient management and taking necessary isolation procedures.

We aimed to determine demographic, clinical and laboratory variables that may be useful in differentiating CCHF from other febrile diseases in patients with suspected CCHF at the time of initial presentation. The study was designed to help clinicians determine optimal strategies for diagnosing CHF and isolating patients with suspected CHF at an early stage.

## Material and methods

### Study design and population

The study was conducted retrospectively among 117 patients admitted to Giresun Training and Research Hospital with preliminary diagnosis of CCHF between September 2021 and October 2023. According to the Ministry of Health's CCHF case management algorithm, hemogram results were evaluated for patients who presented with at least two of the following symptoms: sudden onset fever, headache, generalized body pain, arthralgia, malaise, diarrhea, and signs of bleeding, along with a history of tick bite, living in an endemic area, or travel to an endemic area. Among these patients, patients with a platelet count below 150,000 mm^3^ or a leukocyte count below 4000/mm^3^ were included in the study. The study included adult patients (aged 18 and over) for whom an ELISA for CCHFV IgM or a PCR test for CCHFV RNA was requested at the National Public Health Reference Laboratory using serum material. Seventy-four patients with a definitive diagnosis of CCHF with these tests and 43 patients with negative CCHF tests were divided into two different groups and compared in terms of clinical findings, laboratory findings and outcomes. Demographic data of the patients, presence of tick bite, husbandry, farming, living in endemic area, symptoms, Leukocyte Count (WBC), lymphocyte count, neutrophil count, Hemoglobin (Hb), Hematocrit (Hct), Platelet count (PLT), Lactate Dehydrogenase (LDH), Creatine Kinase (CK), Alanine Aminotransferase (ALT), Aspartate Aminotransferase (AST), C-Reactive Protein (CRP), Procalcitonin (PCT), creatinine, Prothrombin Time (PT), International Normalized Ratio (INR), activated Partial Thromboplastin Time (aPTT), fibrinogen, length of hospital stay, need for intensive care unit, in hospital mortality and 28-day mortality rates were obtained from the electronic hospital record system and compared between the two groups.

### Statistical analysis

IBM SPSS Statistics for Windows version 26.0 (IBM Corp., Armonk, NY, USA) was used for statistical analysis. Mean, standard deviation, percentages and median (minimum‒maximum) were used for descriptive statistics. Normal distribution of quantitative data was tested by Kolmogorov-Smirnov test. In the comparison of quantitative data, independent sample *t*-test was used when the normal distribution condition was met, and Mann–Whitney *U* test was used when this condition was not met. Chi-Square test or Fisher's exact test was used to compare proportions between independent groups. Univariate and multivariate logistic regression analysis were used to identify independent factors to support the diagnosis of CCHF. A multivariate logistic regression model was created with variables with *p*<0.100 in univariate analysis. The area under the Receiver Operating Characteristics (ROC) curve was used to determine the cut-off, sensitivity and specificity values for CRP, procalcitonin, neutrophil count and WBC variables in predicting the diagnosis of CCHF. A significance level of *p*<0.05 was accepted for statistical significance.

## Results

The study included 117 patients with preliminary diagnosis of CCHF. The mean age of the patients included in the study was 55.3 ± 18.1 (19‒94). Seventy (59.8 %) patients were male. In the negative group, 10 patients were diagnosed with sepsis, 5 with malignancy (3 acute myeloid leukemia, 1 colon cancer, 1 myelodysplastic syndrome), 4 with hemorrhagic fever with renal syndrome, 3 with leptospirosis, 3 with COVID-19, 3 with Immune Thrombocytopenic Purpura (ITP), 2 with brucellosis and 1 with febrile neutropenia. Twelve patients did not have a specific diagnosis. These 12 patients whose clinical and laboratory findings improved spontaneously during follow-up was considered as nonspecific viral infection. Sixty-three patients (53.8 %) reported tick bite and 56 (75.7 %) of these patients were in the CCHF group (*p*<0.001). Husbandry, farming, malaise, loss of appetite, nausea-vomiting and living in endemic area were more common in the CCHF group (*p*<0.001). Symptoms of the patients at admission were evaluated; fever, headache, myalgia were more common in the CCHF group (*p*<0.001). Abdominal pain was significantly higher in the non-CCHF group (*p*<0.023). Other demographic data, symptoms and findings are shown in Table 1.

**Table 1:** Demographic and clinical characteristics of patients.

	CCHF (*n* = 74)	Non-CCHF (*n* = 43)	*p*
Age	51.6 ± 15.6 (19‒80)	61.6 ± 20.3(21‒94)	0.004
Gender	M:39 (52.7 %)	M:31 (72.1 %)	0.039
	F:35 (47.3 %)	F:12 (27.9 %)	
Tick bite	56 (75.7 %)	7 (16.3 %)	<0.001
Husbandry	60 (81.08 %)	12(27.9 %)	<0.001
Farming	54 (72.9 %)	12 (27.9 %)	<0.001
Living in endemic area	73 (98.64 %)	24 (55.81 %)	<0.001
Fever	62 (83.78 %)	21(48.83 %)	<0.001
Headache	25 (33.78 %)	5 (11.62 %)	0.008
Myalgia	46 (62.16 %)	13 (30.23 %)	<0.001
Malaise	64 (86.48 %)	22 (51.16 %)	<0.001
Loss of appetite	30 (40.54 %)	8 (18.60 %)	0.015
Nause-vomiting	33 (44.59 %)	11 (25.58 %)	0.041
Abdominal pain	5 (6.75 %)	9 (20.93 %)	0.023
Diarrhea	11 (14.86 %)	2 (4.65 %)	0.09
Hematuria	1 (1.35 %)	1 (2.32 %)	0.695
Petechiae	2 (2.70 %)	0 (0 %)	0.277
Epistaxis	1 (1.35 %)	0 (0 %)	0.444
Gum bleeding	1 (1.35 %)	0 (0 %)	0.444
Uterin/vaginal bleeding	3 (4.05 %)	0 (0 %)	0.181

Cytopenic findings such as low WBC, neutrophil count and PLT were more frequent in the CCHF group (*p*<0.05). AST, ALT, LDH were significantly higher in the CCHF group. Elevated CRP and PCT were significantly higher in the non-CCHF group (*p*<0.05) (Table 2).

**Table 2:** Laboratory results of patients at admission.

	CCHF (*n* = 74)	Non‒CCHF (*n* = 43)	*p*
WBC (×10^9^ cells/L), mean ± SD (min‒max)	2.51 ± 1.15 (0.74‒6.25)	8.84 ± 7.59 (1.09‒29.74)	<0.001
Lymphocyte (×10^9^ cells/L), mean ± SD (min‒max)	0.76 ± 0.44 (0.18‒2.79)	1.17 ± 1.28 (0.22‒6.01)	0.208
Neutrophil (×10^9^ cells/L), mean ± SD (min‒max)	1.53 ± 1.08 (0.3‒5.65)	7.07 ± 6.75 (0.63‒27.75)	<0.001
Hemoglobin (g/dL), mean ± SD (min‒max)	13.8 ± 1.6 (9.6‒19.1)	12.7 ± 2.0 (7.3‒17.4)	0.004
Thrombocyte (×10^9^ cells/L), mean ± SD (min‒max)	78.85 ± 26.86 (18‒128)	107.96 ± 63.38 (10.0‒238.0)	0.021
Creatinine (mg/dL), mean ± SD (min‒max)	0.85 ± 0.32 (0.44‒2.87)	1.70 ± 1.29 (0.58‒5.12)	<0.001
ALT (U/L), mean ± SD (min‒max)	141 ± 315 (13‒2622)	118 ± 214 (7‒1110)	0.033
AST (U/L), mean ± SD (min‒max)	199 ± 283 (13‒1738)	147 ± 293 (12‒1830)	0.001
LDH (U/L), mean ± SD (min‒max)	527 ± 477 (207‒3992)	488 ± 508 (124‒2764)	0.005
CK (U/L), mean ± SD (min‒max)	492 ± 890 (53‒6679)	656 ± 1491 (15‒5709)	0.009
PT, mean ± SD (min‒max)	10.1 ± 2.4 (7.0‒19.90)	12.6 ± 11.1 (7.8‒80.0)	0.102
Fibrinogen, mean ± SD (min‒max)	1 (6.7)	7 (23.3)	0.367
INR, mean ± SD (min‒max)	1.04 ± 0.18 (0.87‒1.7)	1.24 ± 0.93 (0.83‒7.0)	0.017
aPTT (sec), mean ± SD (min‒max)	33.7 ± 8.9 (21.7‒78.0)	35.7 ± 35.30 (20.1‒257)	0.090
CRP (mg/L), mean ± SD (min‒max)	17 ± 27 (1.0‒124.0)	108± 120 (1.0‒434.0)	<0.001
PCT (ng/mL), mean ± SD (min‒max)	0.59 ± 2.37 (0.02‒20.4)	12.7 ± 23.71 (0.01‒100.0)	<0.001

ALT, Alanine Transaminase; aPTT, Activated Partial Thromboplastin Time; AST, Aspartate Transaminase; CK, Creatin Kinase; CRP, C-Reactive Protein; INR, International Normalized Ratio; LDH, Lactate Dehydrogenase; SD, Standard Deviation; WBC, White Blood Cell, PCT, Procalcitonin; PT, Prothrombin Time.

In order to evaluate the role of acute phase reactants and hemogram parameters in predicting the diagnosis of CCHF; WBC, neutrophil count, CRP and PCT values were analyzed by ROC curve (*p*<0.001). The sensitivity, specificity and cut off values of WBC, CRP, neutrophil count and PCT values in predicting CCHF are shown in Table 3. The specificity for cut off values of 2485 mm^3^ for WBC and 970 mm^3^ for neutrophil count were 86 % and 93 %, respectively (AUC = 0.845 [0.762–0.929] and AUC = 0.856 [0.781–0.931]). The sensitivity for cut off values of 48 mg/L for CRP and 0.52 ng/mL for PCT were 90.5 % and 82.4 %, respectively (AUC = 0.808 [0.721–0.896] and AUC = 0.782 [0.644–0.919]).

**Table 3:** ROC analysis for the ability of variables to discriminate CCHF.

	Cut-off	AUC (95 % CI)	Sensitivity	Specificity	*p*
WBC (/mm^3^)	>2485	0.845 (0.762–0.929)	59.5	86.0	<0.001
Neutrophil (/mm^3^)	>970	0.856 (0.781–0.931)	40.5	93.0	<0.001
CRP (mg/L)	<48	0.808 (0.721–0.896)	90.5	53.5	<0.001
PCT (ng/mL)	<0.52	0.782 (0.644–0.919)	82.4	70.8	<0.001

AUC, Area Under Curve; CI, Confidence Interval; CRP, C-Reactive Protein; PCT, Procalcitonin; WBC, White Blood Cell.

A logistic regression model was created with epidemiologic data and symptoms that were significant in favor of CCHF, as well as CRP and PCT variables that showed high sensitivity in ROC analysis. The results of multivariate logistic regression analysis are shown in Table 4. Living in endemic area (OR = 82.648 [5.340–1279.182]), presence of tick bite (OR = 15.845 [2.978–84.314]), fever (OR = 14.512 [2.270–92.776]), CRP below 48 mg/L (OR = 10.976 [1.580–76.226]) and PCT below 0.52 ng/mL (OR = 10.353 [2.064–51.935]) were determined as independent risk factors for CCHF diagnosis.

**Table 4:** Logistic regression analysis for CCHF diagnosis.

	OR (95 % CI)	*p*
Living in endemic area	82.648 (5.340–1279.182)	0.002
Tick bite	15.845 (2.978–84.314)	0.001
Fever	14.512 (2.270–92.776)	0.005
CRP < 48 mg/L	10.976 (1.580–76.226)	0.015
PCT < 0.52 ng/mL	10.353 (2.064–51.935)	0.005

CI, Confidence Interval; CRP, C-Reactive Protein; OR, Odds Ratio; PCT, Procalcitonin.

In hospital mortality and 28-day mortality were higher in the non-CCHF group and this was statistically significant (*p*<0.05). The comparison of intensive care unit need and length of hospital stay in both groups is shown in Table 5.

**Table 5:** Outcomes.

	CCHF (*n* = 74)	Non-CCHF (*n* = 43)	*p*
Intensive care unit admission, *n* (%)	5(6.8 %)	9(2.1 %)	0.023
In hospital mortality, *n* (%)	2(2.7 %)	7(16.3 %)	0.008
28-day mortality, *n* (%)	1(1.4 %)	6(13.9 %)	0.006
Length of hospital stay, mean ± SD (min‒max)	7.9 ± 6.6 (2‒56)	10.0 ± 7.5 (2‒32)	0.236

SD, Standard Deviation.

## Discussion

The differential diagnosis of CCHF is includes a wide range of diseases. Many infectious diseases including other hemorrhagic fever causes, sepsis, febrile neutropenia, malaria, rickettsioses, relapsing fever, Q fever, brucellosis, leptospirosis, viral hepatitis, meningococcemia and non-infectious diseases (ITP and leukemias) should also be kept in mind.^5^^,^^7^^,^^8^

The tests required for the diagnosis of CCHF are performed in national central reference laboratories and results are obtained within days, which delays the diagnosis. This situation may lead to failure in the evaluation of many viral, bacterial and noninfectious diseases in the differential diagnosis, patient management and taking necessary isolation procedures. In our study, we investigated the differentiation of patients with CCHF at admission from those without CCHF based on demographic, clinical findings and laboratory parameters.

Tick bite has an important role in transmission. In various studies, the rate of tick bite was found to be between 57.9 % and 74.2 %.^9^^,^^10^ In our study, this rate was found to be 75.7 % and was significantly more frequent in the CCHF group (*p*<0.001). In our study, as reported in previous publications, the incidence of CCHF was statistically higher in those living in the endemic region and those working in farming and animal husbandry (*p*<0.05).^4^^,^^5^ It is noteworthy that living in an endemic area and tick bite were associated with 82.6 and 15.8-fold risk for CCHF diagnosis in multivariate logistic regression analysis, respectively. Many studies and reviews have reported tick bite and a history of living in or traveling to endemic areas as warning signs for the disease.^4^, ^5^, ^6^, ^7^, ^8^, ^9^ These factors are also included in the probable case definitions for CCHF by the Ministry of Health in our country.^11^ Therefore, it is not surprising that these two variables have the highest independent impact on the diagnosis of CCHF.

Fever, headache, malaise, weakness, anorexia, nausea, vomiting and myalgia are the expected findings of this disease in the early period and were found to be statistically significant in our study.^9^^,^^12^ The presence of fever at admission in 62 out of 74 CCHF-positive patients is a significant finding. Fever typically appears during the pre-hemorrhagic phase of the disease, along with other flu-like symptoms. In our study, symptoms such as headache, fatigue, loss of appetite, nausea-vomiting, and myalgia were statistically more frequent in CCHF cases. However, similar symptoms can be observed in many other diseases in the absence of fever and these clinical findings do not provide important clues to the clinician since they are also seen in many diseases that should be considered in the differential diagnosis of CCHF.^6^^,^^13^ In the multivariate logistic regression analysis, fever was found to be an independent clinical finding for the diagnosis of CCHF (OR = 14.512 [2.270‒92.776]). Considering that some of the diseases in the differential diagnosis are of non-infectious origin, the presence of fever stands out as a key initial finding for CCHF diagnosis, with an odds ratio of 14.5. Abdominal pain was statistically more common in the non-CCHF group. In a previous study, abdominal pain was found to be statistically significant in the group of patients with CCHF who developed bleeding.^14^ These findings suggest that abdominal pain may be significant in the hemorrhagic phase rather than in the early stage of the disease. Since our study aimed to differentiate the disease at the time of presentation, abdominal pain in the early stage should rather suggest other diseases in the differential diagnosis.

In laboratory parameters, as expected, leukopenia, thrombocytopenia and transaminase elevation were more frequent in the CCHF group. CK mean levels were high in both groups, but contrary to other studies, they were significantly higher in the non-CCHF group.^9^^,^^10^ It was thought that the heterogeneous distribution in the non-CCHF group, ranging from severe infections such as sepsis to malignant diseases (acute myeloid leukemia etc.), might further increase the mean value of CK. In our study, creatinine was mostly within normal limits in the CCHF group and the mean value was within normal limits. Creatinine elevation for CCHF has rarely been reported in the literature. This condition has mostly developed in the hemorrhagic period and has been associated with mortality.^15^^,^^16^ Creatinine elevation is not an expected finding among the presenting findings of CCHF patients.^9^^,^^10^^,^^17^ In our study, it was higher and statistically significant in the non-CCHF group.

Endothelial damage developing with increased cytokine release and immune response plays an important role in the pathogenesis of the disease. As a result of tissue and organ damage that develops accordingly, an increase in acute phase reactants may be expected with the contribution of inflammatory processes.^18^^,^^19^ However, since tissue and organ damage has not yet developed in the early period, a lower acute phase reactant response occurs. Therefore, although some increase in CRP is expected in patients with CCHF, in general, lower CRP levels have been reported compared to other diseases in the differential diagnosis.^20^ In addition to studies indicating that the initial CRP value may not be indicative of prognosis in mild cases, there are studies showing that CRP increase is associated with mortality and severity of the disease.^21^, ^22^, ^23^, ^24^ In our study, the CRP value at admission was lower in CCHF patients compared to the other group. It was determined that the CRP value at the time of hospital admission showed a sensitivity of 90.5 % when the cut-off value for predicting the diagnosis of CCHF was below 48 mg/dL. According to this cut-off value, CRP below 48 mg/dL at admission was an independent predictor for the diagnosis of CCHF in logistic regression analysis (OR = 10.976 [1.580‒76.226]). Considering this information, a CRP above 48 mg/dL at admission should be prioritized over other diseases in the differential diagnosis.

Procalcitonin elevation is mostly an indicator of bacterial infections.^25^ There are studies showing that procalcitonin elevation in CCHF patients is associated with disease severity, mortality and bleeding.^14^^,^^26^, ^27^, ^28^ However, there is limited data on the value of PCT in predicting the diagnosis of CCHF. In a study by Kaygusuz et al. comparing CCHF and other diseases in the differential diagnosis, PCT levels were found to be higher in the CCHF negative group.^24^ Similarly, in our study, low PCT levels were found to be associated with the diagnosis of CCHF. This may be related to the fact that a significant proportion of cases in the negative group had sepsis and other bacterial infections. In addition, it was thought that concomitant creatinine elevation in the CCHF-negative group may also contribute to PCT elevation.^29^ When a cut-off value of 0.52 ng/mL was considered as the cut-off value for predicting the diagnosis of CCHF, it was determined that PCT showed 82.4 % sensitivity below this value. According to this cut-off value in logistic regression analysis, PCT below 0.52 ng/mL at admission was an independent predictor for the diagnosis of CCHF (OR = 10.353 [2.064‒51.935]). Based on these data, high PCT levels at admission should primarily direct the clinician to other diseases in the differential diagnosis.

Cytopenias such as leukopenia and thrombocytopenia are prominent findings of CCHF which are thought to develop due to apoptosis in intravascular and lymphoid organs and hemophagocytosis.^30^^,^^31^ In our study, WBC, neutrophil count and platelet count were found to be significantly lower in the CCHF group. ROC analysis was performed to determine the cut off values for WBC and neutrophil count in the diagnosis of CCHF. WBC and neutrophil counts above 2485 mm^3^ and 970 mm^3^, respectively, showed 86 % and 93 % specificity for CCHF. These values were thought to be helpful in excluding the diagnosis of CCHF in clinical practice. Almost all of the patients included in the study had different levels of thrombocytopenia. Therefore, detailed statistical analysis was not performed for the PLT variable since it would provide limited contribution to the clinician in differential diagnosis.

In terms of outcomes, in hospital mortality, 28-day mortality and need for intensive care unit rates were higher in the non-CCHF group (Table 5). This situation reveals that in addition to the importance of identifying CCHF patients, the disease should also be rapidly excluded and other differential diagnoses should be addressed. In a study conducted by Bozkurt, it was reported that only 21 % of the patients diagnosed with CCHF were treated, followed-up and isolated before definitive diagnosis.^32^ The data obtained in our study will contribute to the treatment and follow-up approaches and prognosis, including mortality, by making or excluding the diagnosis of CCHF.

The fact that our study was single-center and retrospective may be considered as limitations.

## Conclusion

Making a rapid prediction based on laboratory and clinical findings ensures both early diagnosis and treatment of the disease and timely isolation procedures. The right approach allows for rapid differential diagnosis with other critical illnesses with completely different treatments that carry a high mortality risk, and is also important in terms of breaking the chain of transmission. In patients living in an endemic area, tick bite and patients with clinical findings compatible with CCHF, easily accessible tests such as WBC, neutrophil count,CRP and PCT will help to diagnose CCHF at the initial admission within the cut offs determined in our study.

## Ethics

This study was approved by the institutional review board at our institution with the document no 25.12.2023/21.

## Authors’ contributions

All authors was responsible for the conceptualization and the development of the study design, coordination of the research activities, and manuscript writing. AMŞ served as the chief investigator, overseeing data collection, analysis, and interpretation. All authors, participated in the writing and revision of the final manuscript.

## Funding

This research did not receive any specific grant from funding agencies in the public, commercial, or not-for-profit sectors.

## Conflicts of interest

The authors declare no conflicts of interest.
